# The Treatment of Glycosuria by Trypsin and Secretin

**Published:** 1907-06-22

**Authors:** L. A. Clutterbuck


### The General Practitioners' Column.

[Contributions to this Column are invited, and if accepted will be paid for.]

### THE TREATMENT OF GLYCOSURIA BY TRYPSIN AND SECRETIN.

By L. A. CLUTTERBUCK, M.D., M.R.C.P.

It has long been recognised that there are cases of glycosuria which depend upon a failure, in some direction, of the functions of the pancreas or its secretions. Possibly such cases are sufficiently common to make it worth while to try the treatment indicated below in every case in which sugar is found in the urine. The patients whose cases are described had both been under treatment in hospital for diabetes, and there is no doubt their condition was not merely a passing phase.

I wish to say a few words with regard to the preparations used. Bayliss and Starling * showed that the secretion of the pancreas was normally called into play by a substance formed in the mucous membrane of the upper parts of the small intestine; and that the absorption of this substance, which they called secretin, into the blood, was followed by the rapid flow of pancreatic juice with a strong amylolytic action. The preparation of secretin I used was supplied by Messrs. Allen and Hanbury. The other substance chiefly employed by me was trypsin; possibly the market preparations of this drug contain other pancreatic products, and not only the proteolytic ferment.

### The First Case.

The first patient, a man aged 49, came under my care at the end of December 1905, having been in hospital, and subsequently at a convalescent home, for some three months between April and July. Sugar was present in his urine during January 1906; and on February 9th a fermentation apparatus, afterwards found to be, in my hands, very unreliable, showed the quantity as 1.13 per cent., which is believed to be much too low. The patient was put on secretin ʒj. twice daily, with no other drug. On February 23rd the sugar in the urine, estimated by Carwardine's saccharometer, was much less than 1 per cent. He was then given enough secretin to last, in the above doses, for one week only. On March 9, the patient having been without secretin for a week, the urine contained 2.5 per cent. of sugar; he was again put on secretin ʒj. twice daily. On March 23rd, and again on April 6th, the sugar amounted to 1.1 per cent., and no change was noticed on May 4th. During the foregoing period the patient was not passing more than four or five pints of urine a day, and did not suffer appreciably from thirst; he was eating gluten bread and abstaining as much as possible from carbohydrate food. I did not see him again till February 1st, 1907, he having had no medicinal treatment in the interval. His urine then contained 5 per cent. (=22 grains per oz.) of sugar; he was put on liquor pancreaticus ʒss. thrice daily, and trypsin gr. j. twice daily. The sugar uninterruptedly diminished until it was less than 1 per cent. on February 22nd, no change having been made in the diet meanwhile.

### The Second Case.

The second patient was a woman aged 62, who came under my care in August 1906, immediately after leaving hospital. She was on a restricted diet, eating no bread untoasted, and little, if any, other starchy or saccharine food. On September 4th and October 5th her urine showed traces of sugar. On the latter date she was given tinct. opii ♏.5, thrice daily. On October 12th the urine readily reduced Fehling's solution, and she was then given trypsin gr. 2, thrice daily, but no other drug. On October 19th the urine did not reduce Fehling's solution; the trypsin was discontinued. On October 26th there was a slight reduction of Fehling's solution. She was put on trypsin as before, and was told to relax slightly her dietetic restrictions. On November 2nd she had been taking two slices of untoasted bread and a potato daily for a week; the urine did not reduce Fehling's solution, and the trypsin was again withdrawn. On November 7th the urine reduced Fehling's solution, and the sugar, estimated with a safranin solution, amounted to between 0.5 and 1 per cent. Subsequent examinations of the urine in November and at the beginning of December did not detect sugar, but on December 14th the urine again reduced Fehling's solution, and trypsin gr. 1 daily was ordered. On December 21st the urine still reduced Fehling's solution, so the trypsin was raised to gr. 1 twice daily. On January 11th, 1907, there was still a trace of sugar in the urine, but specimens examined on January 18th (when the patient was passing 2½ pints daily), on four dates in February, and on March 1st, did not reduce Fehling's solution. All this time the patient had been taking trypsin gr. 1 twice daily. On March 1st all restrictions as to diet were withdrawn, and the patient given trypsin gr. 1 thrice daily. Sugar appeared in the urine on March 8th, so the trypsin was doubled—gr. 2, thrice daily—the patient to remain on full diet. On March 15th sugar was still present in the urine, so she was told to return to the previous diet—a little untoasted bread and potato daily—and was given liquor pancreaticus ʒj. and trypsin gr. 1, thrice daily. On March 22nd and April 5th the urine did not reduce Fehling's solution.

In the qualitative tests for sugar equal quantities of urine and Fehling's solution were used. In the quantitative tests Carwardine's saccharometer was used, except where otherwise stated. It will have been seen that, in the first case, the administration of secretin was on at least two occasions followed by a diminution of sugar in the urine, while a similar result followed the use of liquor pancreaticus and

* "Journal of Physiology," September 1902.

trypsin, when the amount of sugar was as much as 5 per cent. In the second case, the use of trypsin alone was followed on three occasions—October 19th and November 2nd, 1906, and January 18th, 1907—by complete disappearance of sugar from the urine, even with some latitude in regard to diet. A similar result followed the use of trypsin and liquor pancreaticus together on one occasion—March 22nd, 1907; for a period of seven weeks, from January 11th to March 1st, during which the patient was taking trypsin gr. 1 twice daily, sugar was continuously absent from the urine; while the withdrawal of the drug was on two occasions—October 26th and November 7th—immediately followed by the reappearance of sugar in the urine. The above facts tend to show that some mild cases of glycosuria can be completely controlled by the use of trypsin, and that it is necessary that the drug should be given more than once a day, preferably three times, in relation to the chief meals of the day.

